# Is my patient fit to be discharged? – A numerical answer to a difficult question

**DOI:** 10.1186/1865-1380-8-S1-P9

**Published:** 2015-04-22

**Authors:** Pragya Mallick, Balaji Natarajan, Jassica Adaikalanadhan, Tausif A Thangalvadi, Parivalavan Rajavelu

**Affiliations:** 1 Sundaram Medical Foundation, Chennai

## Objectives

Discharging patients from the Emergency Department (ED) poses a huge conundrum. Limited time of contact, emergent nature of cases, shortage of inpatient beds and cost factors contribute to premature discharge decisions that can have grave consequences. There is need for a reliable and objective tool to help safely discharge patients from the ED. The “SMF ED safe discharge score”was proposed as one such tool. This study aims to determine a correlation between clinical judgement and the Sundaram Medical Foundation (SMF) ED safe discharge score and its relevance to the safe discharge of ED patients.

## Methodology

This was a prospective, observational study done in the ED of Sundaram Medical Foundation, Chennai, over a period of 10 days in August 2014. The proposed score used the acronym “DISCHARGED” with 10 components namely Disability, Investigations, Saturation (O2), Co-morbidities, Heart rate, Average SBP, Referral, Geriatrics, ED events and Domestic factors, each graded 0, 1 and 2. Once the patient was discharged as per the Emergency Physician’s clinical judgment, his/her score was entered out of 20. Follow up was done after 10 days of first ED visit. Inclusion criteria: all patients getting discharged from the adult ED (age>17 yrs). Exclusion criteria: Referral to another hospital, reports review, injection patients, admitted or expired patients, absconded/discharged AMA. Outcome measures: revisit for the same complaint- planned or unplanned, to ED, specialist or other hospital, admission on revisit or death.

## Results

The study enrolled 141 patients. Total number of patients followed up was 100. 41 were lost to follow up. There were zero deaths in the study group. The average DISCHARGED score was 2.7 with 10 as maximum and 0 as minimum. 51 patients did not revisit and had an average score of 2.1. 49 patients revisited (avg score 3.3). 44 were planned revisits to Specialist (31), report review (5), other hospitals (8) with average scores being 3.2, 3.4 and 3.3 respectively. 6 got admitted (average score 4.5) and 38 went home (average score 3.2). 5 had unplanned visits (average score 3) of whom 1 needed admission. Only 7 out of 100 patients needed admission with average score of 4.7. Using scores ≥ 6 and ≤ 1, the sensitivity was 100% and specificity 88.9% for safe discharge. The ED events (chief complaints) contributed maximally to the score (47.5%), followed by specialist referral (27%) and investigations (22%).

## Conclusion

SMF ED safe discharge score correctly predicts admission and revisits in discharged patients. It can be used as an adjunct tool to support clinical judgement for safe discharge of patients from ED.

